# Supplementary material for: Differences in Extracellular NAD+ and NMN Metabolism on the Surface of Vascular Endothelial Cells
Source: Biology (Basel). 2022 Apr 27;11(5):675. doi: 10.3390/biology11050675 (PMC9137893; doi:10.3390/biology11050675)
Supplement: Supplementary file 1 [file biology-11-00675-s001.zip › biology-1648361-supplementary.pdf]

SUPPLEMENTARY MATERIAL

Differences in extracellular NAD+ and NMN metabolism on the surface of vascular endothelial cells

Patrycja Jablonska[1], Paulina Mierzejewska[1], Marta Tomczyk[1], Patrycja Koszalka[2], Marika Franczak[1], Ada Kawecka[1], Barbara Kutryb-Zajac[1], Alicja Braczko[1], Ryszard T. Smolenski[1] and Ewa M. Slominska[1] *

[1] Department of Biochemistry, Medical University of Gdansk, 80-211, Gdansk, Poland; patrycja.jablonska@gumed.edu.pl (P.J.); paulina.mierzejewska@gumed.edu.pl (P.M.); marta.tomczyk@gumed.edu.pl (M.T.); kaweckaada@gumed.edu.pl (A.K.); b.kutryb-zajac@gumed.edu.pl (B.KZ.); alicja.braczko@gumed.edu.pl (A.B.); marika.franczak@gumed.edu.pl (M.F.); ryszard.smolenski@gumed.edu.pl (R.T.S.)

[2] Institute of Medical Biotechnology and Experimental Oncology, Laboratory of Cell Biology and Immunology, Intercollegiate Faculty of Biotechnology University of Gdansk and Medical University of Gdansk, 80-211, Gdansk, Poland; patrycja.koszalka@gumed.edu.pl (P.K.);

SUPPLEMENTAL FIGURES

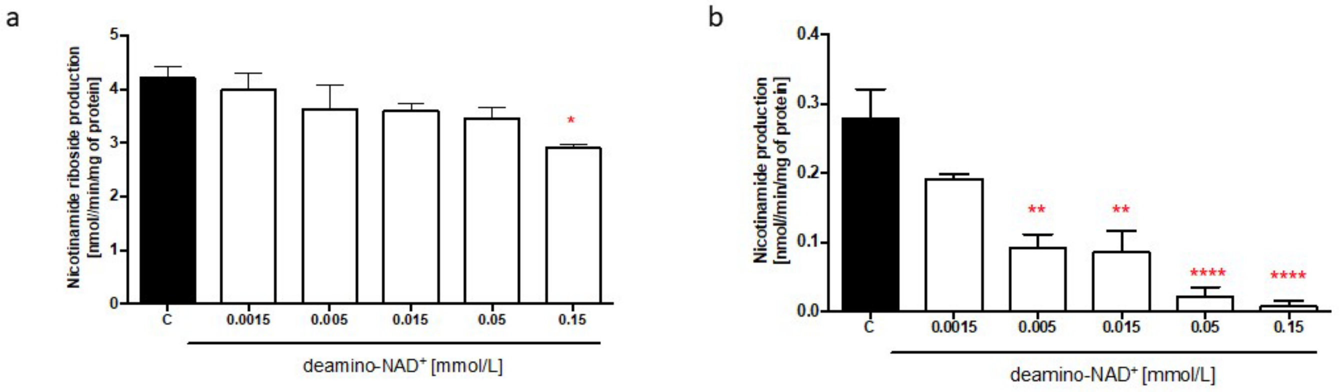

Figure S1. Determination of the dose inhibiting the metabolism of NAD and NMN by deamino NAD. The effect of deamino-NAD+ (nicotinamide hypoxanthine dinucleotide sodium salt) on nicotinamide (Nam) and nicotinamide riboside (NR) production during 120 min co-incubation with nicotinamide mononucleotide (NMN) on Eahy.926 cells. Results are shown as mean +- SEM, n=3-5, ****p<0.0001; **p<0.01; *p<0.05

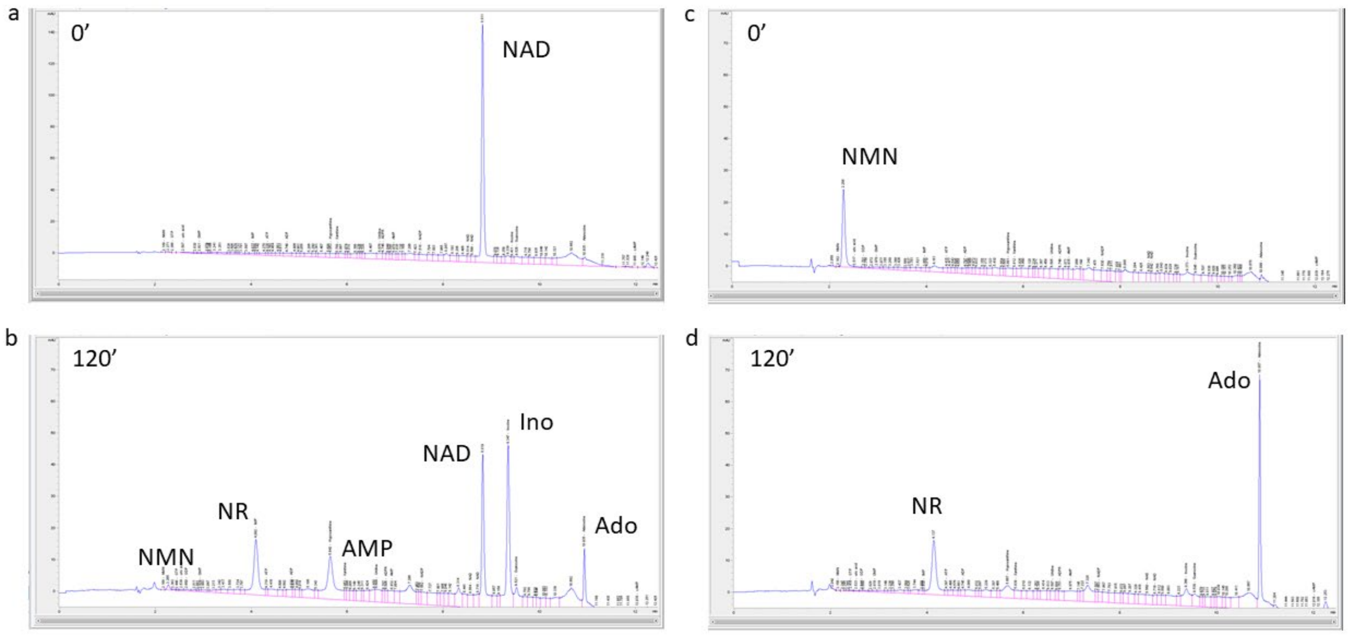

Figure S2. Examples from the chromatographic analysis. NAD hydrolysis at time 0' (Figure S2a) and after 120' minutes of incubation (Figure S2b) on PIEC CD73 cells. NMN hydrolysis at time 0 '(Figure S2c) and after 120' minutes of incubation (Figure S2d) on PIEC CD73 cells**.**
